# *Mycobacterium tuberculosis* DNA Detection Using Surface Plasmon Resonance Modulated by Telecommunication Wavelength

**DOI:** 10.3390/s140100458

**Published:** 2013-12-27

**Authors:** Shih-Hsiang Hsu, Yan-Yu Lin, Shao-Hsi Lu, I-Fang Tsai, Yen-Ta Lu, Hsin-Tsung Ho

**Affiliations:** 1 Department of Electronic Engineering, National Taiwan University of Science and Technology, No. 43, Sec. 4, Keelung Rd., Taipei 10607, Taiwan; E-Mails: shsu@mail.ntust.edu.tw (S.-H.H.); a.j.lin26@gmail.com (Y.-Y.L.); m10002303@mail.ntust.edu.tw (S.-H.L.); 2 Department of Medical Research, Mackay Memorial Hospital, Taipei 25160, Taiwan; E-Mail: m9209001@gmail.com; 3 Division of Chest Medicine, Department of Internal Medicine, Mackay Memorial Hospital, Taipei 10449, Taiwan; E-Mail: ytlhl@ms2.mmh.org.tw; 4 Department of Medicine, Mackay Medical College, 25245, New Taipei City, Taiwan; 5 Department of Laboratory Medicine, Mackay Memorial Hospital, Taipei 25160, Taiwan

**Keywords:** *Mycobacterium tuberculosis*, surface plasmon resonance, wavelength modulation

## Abstract

A surface plasmon resonance sensor for *Mycobacterium tuberculosis* (*MTB*) deoxyribonucleic acid (DNA) is developed using repeatable telecommunication wavelength modulation based on optical fiber communications laser wavelength and stability. *MTB* DNA concentrations of 1 μg/mL and 10 μg/mL were successfully demonstrated to have the same spectral half-width in the dip for optimum coupling. The sensitivity was shown to be −0.087 dB/(μg/mL) at all applied telecommunication wavelengths and the highest sensitivity achieved was 115 ng/mL without thiolated DNA immobilization onto a gold plate, which is better than the sensor limit of 400 ng/mL possible with commercial biosensor equipment.

## 1. Introduction

*Mycobacterium tuberculosis* (*MTB*) is the causative agent of human tuberculosis, a disease that causes millions of deaths every year. Most *MTB*-related deaths could be prevented by early diagnosis and treatment. *MTB* diagnosis is traditionally conducted using smear microscopy and culture methods. However, the former has lower sensitivity and the latter requires about six to eight weeks to process preliminary test reports [1]. *MTB* deoxyribonucleic acid (DNA) detection in clinical specimens such as the sputum and blood from patients suffering from acute pulmonary tuberculosis [2] is reliable because of the stability of DNA. In addition, the long half-life of the DNA pattern, such as insertion sequence (IS) 6110, a DNA sequence specific for *MTB* was typically used to identify the reactivation and track *MTB* transmission [3]. Specific DNA sequence amplification using polymerase chain reaction (PCR) is a sensitive method for mycobacterial detection, but false-positive results could be generated due to amplified DNA contamination from the PCR laboratory [4]. Moreover, this technique requires expensive reagents, testing devices and well-trained personnel. It is necessary to search for more efficient diagnostic methods. Surface plasmon resonance (SPR) sensors have made significant progress in both technical and application aspects mainly because of their instant detection and label-free features. The analyzed analyte and its corresponding binding reaction with the receptor could be instantly detected [5,6].

SPR applications included bacteria, viruses, toxins, allergens, and biomedical analytes besides environmental pollutants [7,8]. Because the infrared light has a longer penetration depth of the surface plasmon than visible light, SPR has been utilized to study living cells and the analyses of cholesterol penetration into membrane and transferin-induced clathrin-mediated endocytosis were demonstrated [9,10]. The relatively large penetration depth of the surface plasmon into a dielectric medium, a few microns in the infrared range, is of the order of the cell height and beneficial in studying cell cultures. The long-wavelength surface plasmon penetration even possibly senses the whole cell volume. SPR penetration depth and propagation length showed around 1 μm and 30 μm, respectively, for the interfaces of Au/water and ZnS/Au/water at a 1,550-nm wavelength [9]. The ability to detect SPR at varying wavelengths and/or varying angles allows “tuning” the surface plasmon resonance to any desired spectral range in order to achieve the highest sensitivity. Since the moving angle repeatability was limited by the optical or mechanical encoder, the telecommunication wavelengths own the wavelength accuracy and will be applied to the SPR for precise wavelength tuning control.

## 2. Design and Simulation

The spectral SPR incident angle is typically set above the critical angle to achieve total internal reflection (TIR). For sharper reflectance from SPR wavelength modulation the minimum reflectance [11], occurring near the resonance angle from SPR angular modulation, was demonstrated as the maximum sensing sensitivity for wavelength variation under constant surrounding analyte.

The angle modulation characterization is not distinguished enough for *MTB* DNA compared with DI water for a small amount of μg/mL due to the existing stepper motor resolution. Therefore, the spectral SPR was characterized at the deionized (DI) water resonance angle of 61.9° for the wavelength modulation.

The Kretschmann structure [11] was utilized for the SPR simulation carried out using the commercial software Matlab to detect *MTB* DNA. The theoretical calculation demonstrated that longer wavelengths will sharpen the reflectance curves in angled modulation for lower SPR sensing sensitivity [12,13]. However, this recent development in fiber optics dramatically increases the transmission capacity and also permits small footprint integrated photonic components/subsystems. Therefore, a 1,550-nm telecommunication wavelength was chosen to detect DI water, acting as the simulation basis for *MTB* DNA biosensing. The real and imaginary parts of complex refractive substance indices are listed in Table 1 at 1,550-nm wavelength [14]. The gold characteristics on complex refractive indices increase with longer wavelengths in the telecommunication range [15].

**Figure 1.**
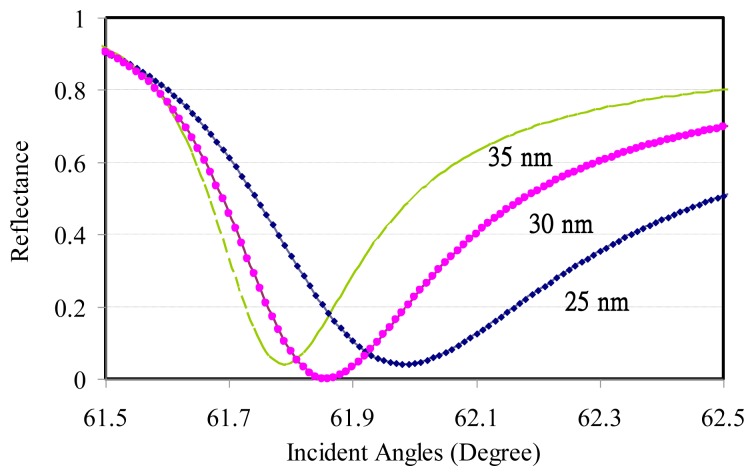
Gold thickness effect on the SPR reflectance for DI water sensing at 1,550-nm wavelength.

Due to the limited information regarding the analyte refractive index variation with the operating wavelengths, only the SPR reflectance sensing of DI water was calculated in the angled modulation to reinforce the test setup. Three different gold film thicknesses were modulated using the incident angles at a 1,550-nm transverse-magnetic (TM) operating wavelength. The dip angles were increased with thinner gold thickness, as shown in Figure 1. The related simulation parameters are listed in Table 2.

The SPR performance is heavily related to the surface plasmon effective index, which is severely affected by the immobilization between the analyte and gold metal layer. Therefore numerous strategies for the immobilization were developed for different types of recognition elements including the proteins, peptides, DNA and more complex natural products [16]. The 5′-thiol end DNA prepared in autoclaved DI water was immobilized onto the pre-cleaned gold surface for 8,500 s at 25 °C and the binding of these molecules was successfully monitored using SPR technology [17]. Glucose biosensing was intentionally used for comparison with the *MTB* DNA on the surface plasmon wave. However, the typical protocol involves utilizing the biomolecule to immobilize a desired surface for analyte sensing, such as glucose oxidase (GOD) for glucose. Here, we planned to utilize the telecommunication wavelength modulation to repeatedly demonstrate the highest sensitivity for *MTB* DNA and glucose without immobilization onto the gold plate.

## 3. Experiment and Results

### 3.1. Reagents and Oligonucleotides

*MTB* DNA was prepared by constructing five representative *MTB* genes (IS6110, 16S ribosomal RNA, 85B, Rv3130c and Rv3133c) into a pUC57 vector. The *MTB* DNA length was 3,133 base pairs. The recombinant *MTB* plasmids were purified using the Qiagen plasmid purification kit (Qiagen, Valencia, CA, USA) according to the manufacturer's instructions and quantitated using NanoDrop™ 2000c (Thermo Fisher Scientific, Wilmington, DE, USA). Chemicals were taken at analytical reagent grade. Distilled water (18.2 MΩ) was used throughout these experiments.

### 3.2. Apparatus

It is necessary to clean the prism before metal deposition. The prism is first placed into an ultrasonic cleaning machine where acetone and 70% ethanol are utilized separately for a period of 10 min. To ensure that the prism surface is free from impurities, residual water and solvents, a nitrogen gun was then used to carefully blow-dry the water vapor off of the prism. Right after cleaning was completed, the prism was sent into the e-beam evaporator for Cr and Au deposition, at thicknesses of 3 nm and 30 nm, respectively. Cr metal was supposed to serve as an adhesive layer. The deposited prism surface was adjusted to an appropriate angle in the deposition chamber so that the metal coverage could be uniform. The experimental resonance angle for DI water was 61.9°, virtually identical with the theoretical calculation, 61.86°, at 30-nm thick gold layer. More glucose concentrations of 100 mg/mL and 200 mg/mL were characterized on the angular modulation.

**Figure 2.**
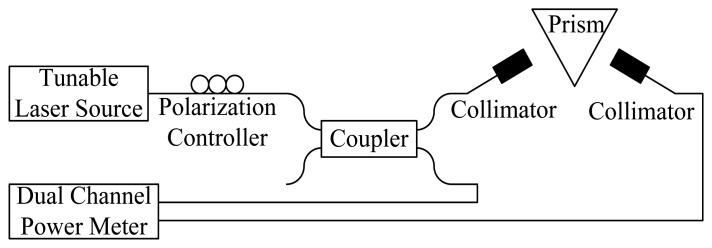
The testing setup for SPR wavelength modulation.

Since the Santec ECL-210 tunable laser source (TLS) could achieve a wavelength repeatability of 0.01 nm, the wavelength modulation was utilized to improve the SPR characterization accuracy. After the incident angle was fixed at 61.9° from the angle modulated SPR, the telecommunication wavelengths were executed using the TLS and modulated at a 10-nm increase from 1,510-nm to 1,590-nm wavelengths. The coupling strength between the analytes and surface plasmon could be observed by adjusting the wavelengths. To ensure the experimental accuracy couplers should be used to monitor the input and output power using a dual-channel power meter, as shown in Figure 2.

**Figure 3.**
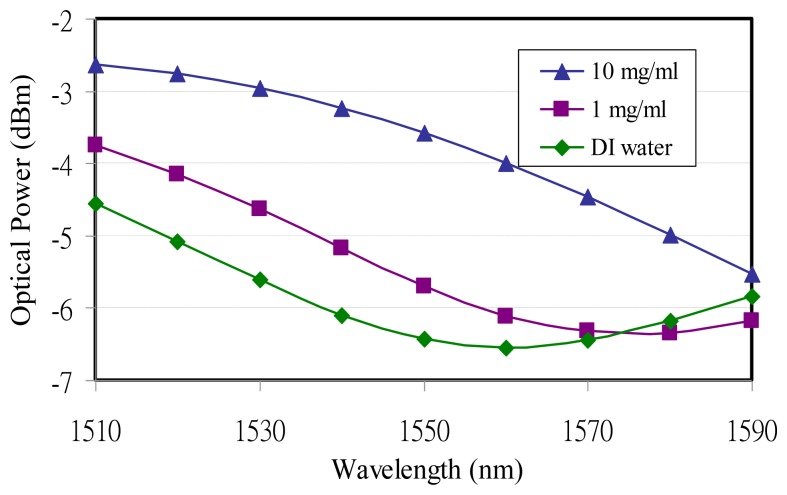
SPR wavelength modulation for glucose concentrations.

An experiment conducted with wavelength modulation was also utilized to detect different glucose concentrations. The TLS wavelength was still tuned from 1,510 nm to 1,590 nm with a 10-nm increase at a resonance angle of 61.9°. The experimental data showed that the resonance wavelengths increased with higher glucose concentrations. The resonance wavelengths occurred at 1,560 nm and 1,580 nm for DI water and 1 mg/mL, milligrams per deciliter (100 mg/dL ≈ 0.1%), concentration, respectively. The resonance wavelength for 10 mg/mL (1,000 mg/dL ≈ 1%) glucose concentration could not be observed due to the TLS wavelength limitation and was expected to be more than 1,590 nm, as shown in Figure 3. Due to the strong surface plasmon guidance the optical power from SPR reflectance increased with a higher glucose concentration.

**Figure 4.**
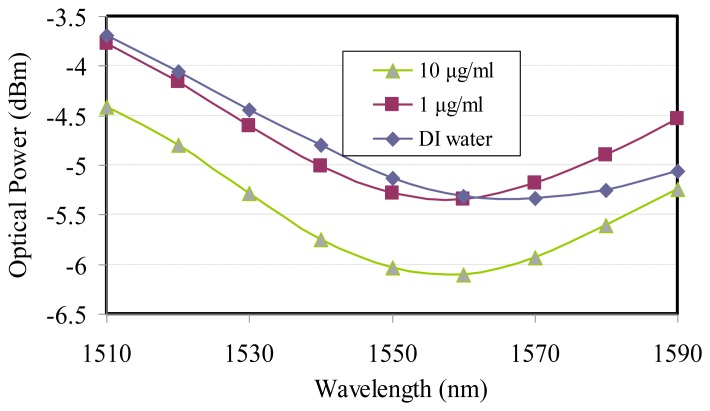
SPR wavelength modulation for *MTB* DNA concentrations.

Additionally, the SPR biosensor modulated using the telecommunication wavelength, the same as the glucose characterization, also successfully detected 1-μg/mL and 10-μg/mL concentrations of the *MTB* DNA, which can be transferred to 0.319 and 3.19 μM, respectively, and were more sensitive than the piezoelectric TB DNA-based biosensor [18], as shown in Figure 4. The data demonstrated that the resonance wavelength of 1,560 nm was applied to two different *MTB* DNA concentrations besides sharing the same spectral half-width of the wavelength dip for the optimum coupling. The optical power from SPR reflectance decreased with higher *MTB* DNA concentration because of the strong surface plasmon guidance.

Due to the limited supply of *MTB* DNA a few data points were demonstrated on the wavelength modulation. In the spectral SPR for *MTB* DNA, the optical power variation was chosen as 1,550-nm instead of the resonance wavelength of 1,560 nm, in which the DI water crossed over 1 μg/mL *MTB* DNA. A similar situation could be applied to the glucose sensitivity characterization.

## 4. Discussion

As for the DI water curve variation with the wavelength modulation for the glucose and *MTB* DNA concentrations, the pH value might be the main reason causing the resonant wavelength shift. DI water is not a good buffer because it does not withstand pH changes. In Figures 3 and 4 the reflective optical power for DI water was different and came from TLS input power adjustment. However, the modulation depths from DI water were all around 2 dB for glucose and *MTB* DNA, which means that the pH variation only has an effect on the resonance wavelengths instead of the reflective SPR curve.

The resonance locations from the wavelength modulation varied with different glucose concentrations, as shown in Figure 3. The longer wavelength is accompanied by higher glucose concentrations. On the other hand the wavelength modulation on *MTB* DNA demonstrated that the resonance wavelengths would not shift when the *MTB* DNA concentration varied, as shown in Figure 4.

**Figure 5.**
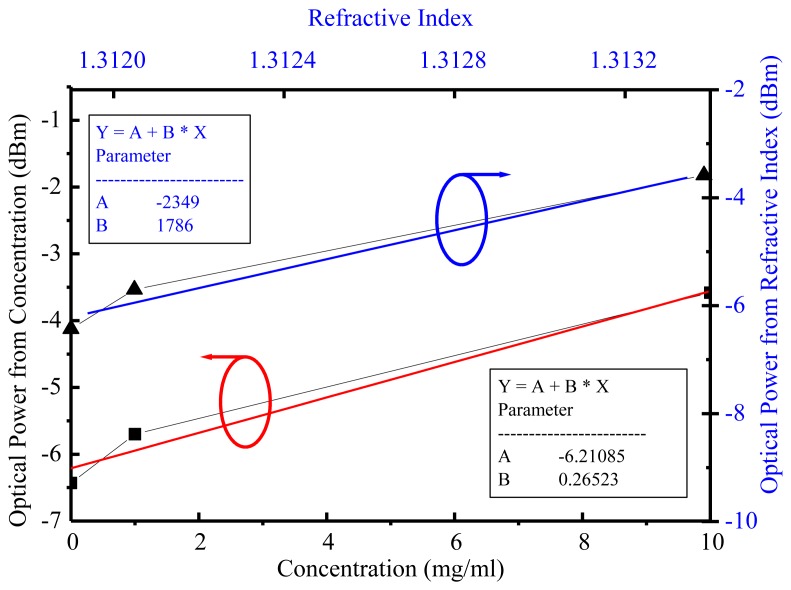
At the wavelength of 1,550 nm, the glucose sensor sensitivity was 0.26 dB/(mg/mL) and 1,786 dB/RIU, respectively, for concentration and refractive index.

Through the theoretical and experimental approaches the SPR reactions on glucose and *MTB* DNA with different detected analytes were investigated. For glucose without immobilization the optical power at a 1,550-nm wavelength was taken for sensitivity analyses on both refractive index [19] and concentration for DI water, 1 mg/mL, and 10 mg/mL. The experimental data demonstrated the glucose refractive index sensitivity and sensing limitation were 1,786 dB/RIU and 5.6 × 10^−6^ RIU, respectively, while 0.01-dBm resolution and accuracy from the optical power meter was achievable. In a similar way the glucose concentration sensitivity was 0.26 dB/(μg/mL) and sensing limitation was 38 μg/mL at a 1,550-nm wavelength, as shown in Figure 5.

For *MTB* DNA without immobilization the same spectral line width shown in Figure 4 implied a linear slope between the concentration and reflective power were the same for all applied telecommunication wavelengths because the surface plasmon effective index did not disperse with the wavelength and the attenuation coefficient (*γ*) will get a constant value at the longer wavelength, as shown in the following equation [16]:

(1) $$\Delta \lambda_{\overset{1}{\;}/\underset{2}{\;}}=\frac{4\gamma }{\left|\frac{dn_{p}}{d\lambda }\sin\theta -\frac{dn_{ef}^{SP}}{d\lambda }\right|}$$

where *θ* is the incident angle. $\frac{dn_{p}}{d\lambda }$is the dispersion of the prism and $\frac{dn_{ef}^{SP}}{d\lambda }$is the dispersion of the effective index of the surface plasmon.

**Figure 6.**
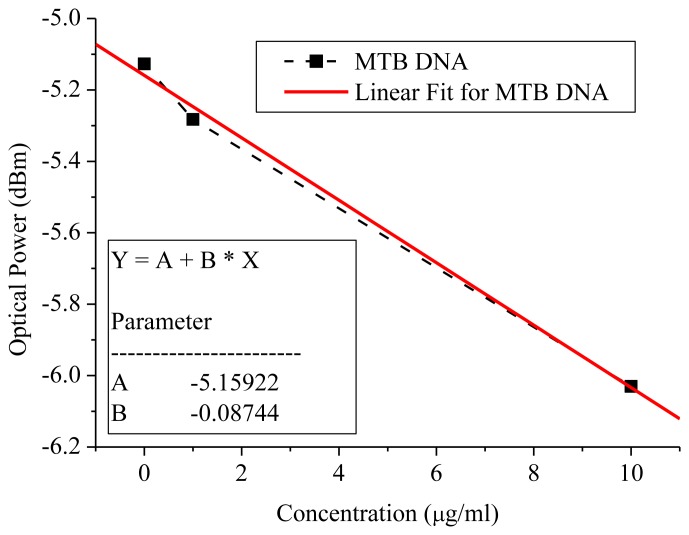
At the wavelength of 1,550 nm, the *MTB* DNA sensor sensitivity was 0.087 dB/(μg/mL).

The linear slope at a 1,550-nm wavelength was demonstrated to be −0.087 dB/(μg/mL), which was the same in the remaining wavelengths, as shown in Figure 6. The 0.01-dBm resolution from the optical power **meter** is typical. Therefore the *MTB* DNA concentration sensing limitation could achieve 115 ng/mL better than the sensor limit of 400 ng/mL from the commercial Thermo Fisher Scientific Nanodrop 2000c micro-volume spectrophotometer equipment with a price of around 10,000 US dollars, as shown in Figure 6.

The highest sensitivity difference between *MTB* DNA and glucose shows that the surface plasmon confinement is analyte related. Because of the surface plasmon guidance, the sensitivity with a negative sign represents the surface plasmon will suffer a higher optical loss in lower *MTB* DNA concentrations and the positive sensitivity in glucose shows a higher optical loss in higher concentrations. After the immobilization was applied the surface plasmon wave propagation would be more efficient and a higher sensing limit than ng/mL was expected [17].

## 5. Conclusions

Because the telecommunication wavelength possesses high precision and stability control in optical fiber communications, the SPR was then modulated using 1,550-nm range wavelengths and *MTB* DNA and glucose analytes were successfully detected without immobilization. These experiments illustrate that a difference exists for surface plasmon waves between *MTB* DNA and glucose. The *MTB* DNA with lower concentrations suffered higher optical loss with the surface plasmon wave, which was applied to glucose with higher concentrations. The highest sensitivity was 5.6 × 10^−6^ RIU for glucose and 115 ng/mL for *MTB* DNA, which is better than the sensor limit of 400 ng/mL obtained from the commercial Thermal Fisher Scientific equipment, the NanoDrop 2000c spectrometer.

## Figures and Tables

**Table 1.** The real and imaginary parts of complex refractive substance indices of DI water and gold material at a 1,550-nm wavelength.

	**Real**	**Imaginary**
DI water	1.318	9.8265 × 10^−5^
Au	0.55	11.5

**Table 2.** Changes in related parameters with different gold film thickness.

**Gold Thickness**	**Dip Angle**	**Reflectance**
25 nm	61.98°	0.041516
30 nm	61.86°	0.000143
35 nm	61.79°	0.038791
